# Impact of Histopathological Response on Outcomes After Surgical Resection Following Carbon‐Ion Radiotherapy for Pancreatic Cancer With Arterial Involvement

**DOI:** 10.1002/ags3.70259

**Published:** 2026-08-02

**Authors:** Kenichiro Araki, Norio Kubo, Yuhei Miyasaka, Hayato Ikota, Ryo Muranushi, Mariko Tsukagoshi, Takamichi Igarashi, Masahiko Okamoto, Tatsuya Ohno, Ken Shirabe

**Affiliations:** ^1^ Division of Hepatobiliary and Pancreatic Surgery, Department of General Surgical Science Gunma University Graduate School of Medicine Maebashi Gunma Japan; ^2^ Department of Radiation Oncology Gunma University Graduate School of Medicine Maebashi Gunma Japan; ^3^ Gunma University Heavy Ion Medical Center Maebashi Gunma Japan; ^4^ Clinical Department of Pathology Gunma University Hospital Maebashi Gunma Japan; ^5^ Department of Radiation Oncology, International Medical Center Saitama Medical University Saitama Japan

**Keywords:** arterial involvement, carbon‐ion radiotherapy, multidisciplinary treatment, pancreatic cancer, surgical resection

## Abstract

**Aim:**

Carbon‐ion radiotherapy (CIRT) provides superior dose distribution and higher biological effectiveness than conventional X‐ray radiotherapy and has emerged as a promising component of multidisciplinary treatment for advanced pancreatic ductal adenocarcinoma (PDAC). However, evidence regarding surgical resection after CIRT remains limited. In this study, we aimed to evaluate the feasibility, histopathological therapeutic response, and oncological outcomes of pancreatic resection after CIRT‐based multimodal treatment.

**Methods:**

We retrospectively analyzed 12 patients with borderline resectable pancreatic cancer with arterial involvement (BR‐A) or unresectable locally advanced PDAC (UR‐LA), who underwent surgical resection following CIRT at our institution between January 2016 and June 2025. The surgical outcomes, perioperative morbidity, pathological therapeutic responses, and survival outcomes were assessed.

**Results:**

Surgical resection after CIRT was feasible in all patients. The procedures included pancreaticoduodenectomy (*n* = 5), distal pancreatectomy (*n* = 2), and distal pancreatectomy with celiac axis resection (*n* = 5). R0 resection was achieved in 11 patients (92%) and no pathological lymph node metastasis was identified. A marked histopathological response (Evans Grades III–IV) was observed in 83% of patients. No local recurrences were observed in the CIRT irradiation field. The 3‐year progression‐free survival and overall survival (OS) rates calculated from treatment initiation were 74.1% and 79.5%, respectively, whereas the corresponding 3‐year recurrence‐free survival and OS rates calculated from surgical resection were 47.6% and 50.8%, respectively.

**Conclusions:**

Surgical resection following CIRT‐based multimodal treatment is feasible and safe, achieving a favorable histopathological response and durable local control in selected patients with PDAC with arterial involvement.

## Abstract 2

Carbon‐ion radiotherapy (CIRT)‐based multimodal treatment followed by surgical resection was feasible and safe in selected patients with borderline resectable or unresectable locally advanced pancreatic cancer with arterial involvement. Surgical resection achieved a high R0 resection rate (92%), remarkable histopathological responses (Evans Grades III–IV, 83%), and durable local control without in‐field recurrence. These findings support the potential role of CIRT as a key component of multidisciplinary treatment to facilitate conversion surgery in advanced pancreatic cancer.

## 1 Introduction

Pancreatic cancer is an increasingly prevalent malignancy and has become the third leading cause of cancer‐related deaths, with a 5‐year survival rate of approximately 13%, underscoring its extremely poor prognosis [1]. Early detection of pancreatic cancer remains challenging, and only a limited proportion of patients are diagnosed at a resectable stage. Consequently, several patients present with tumors that are in contact with or invade major arteries at the time of diagnosis. Borderline resectable pancreatic cancer with arterial involvement (BR‐A) and locally advanced unresectable pancreatic cancer (UR‐LA) remain major therapeutic challenges because conversion surgery after induction therapy is limited by insufficient local control and marginal R0 resection rates [2].

Recent advances in multimodal treatment strategies, including chemotherapy and chemoradiotherapy, have led to improved outcomes in patients with pancreatic cancer. Surgical resection following chemoradiation has emerged as a feasible option in carefully selected patients [3, 4, 5, 6, 7]. Nevertheless, the pancreas is anatomically adjacent to several radiation‐sensitive organs, including the stomach, duodenum, liver, kidneys, and intestines, which often limits the deliverable radiation dose with conventional X‐ray radiotherapy, even when advanced precision techniques are employed. Consequently, durable local tumor control in patients with locally advanced pancreatic ductal adenocarcinoma (PDAC) remains suboptimal with standard X‐ray radiotherapy.

Carbon‐ion radiotherapy (CIRT) provides high relative biological effectiveness and favorable dose distribution, enabling strong local tumor control with acceptable toxicity. Since 2013, our institution has incorporated CIRT as a key component of the multidisciplinary treatment of advanced pancreatic cancer. Since the establishment of a CIRT protocol for UR‐LA pancreatic cancer (dose: 55.2 Gy in 12 fractions), subsequent clinical outcomes have demonstrated favorable local control, with a 2‐year local control rate of 76.1% [8]. In a previous phase I study, Shinoto et al. [9] reported that preoperative CIRT combined with chemotherapy followed by surgical resection for resectable pancreatic cancer was feasible, with no unexpected adverse events, and achieved a favorable 5‐year overall survival (OS) rate of 52%. However, evidence regarding CIRT followed by surgical resection for advanced pancreatic cancer with arterial involvement remains scarce, and no studies have specifically assessed the histopathological treatment response, which is a key determinant of R0 resection and local disease control. Therefore, in this study, we aimed to evaluate the feasibility and oncological validity of surgical resection after CIRT for pancreatic cancer with arterial involvement at our institution.

## 2 Materials and Methods

### 2.1 Study Design and Patient Selection

Among 241 consecutive patients who underwent pancreatectomy for pancreatic ductal adenocarcinoma at Gunma University Hospital between January 2016 and June 2025, 13 who underwent surgical resection following CIRT were retrospectively analyzed. One patient who underwent completion pancreatectomy after CIRT for recurrent pancreatic cancer was excluded. The clinical background, operative findings, postoperative outcomes, and pathological therapeutic responses were reviewed.

The oncological outcomes of patients who underwent conversion surgery were evaluated in terms of progression‐free survival (PFS) and OS calculated from the initiation of initial treatment, as well as recurrence‐free survival (RFS) and OS calculated from the date of surgical resection. In addition, during the same study period, 80 patients diagnosed with BR‐A or UR‐LA pancreatic cancer who were treated with CIRT combined with systemic chemotherapy without subsequent surgical resection were identified. The PFS and OS calculated from the initiation of treatment were compared between patients who underwent surgical resection and those who did not to assess the impact of conversion surgery following CIRT‐based multimodality treatment. The study protocol was approved by the Institutional Review Board of our hospital (approval number: HS2025‐251).

Surgical resection was performed in 12 patients (BR‐A, 3; UR‐LA, 9). The operative outcomes after CIRT or surgical resection were compared with those of other pancreatectomies performed during the same period. The RFS and OS were estimated using the Kaplan–Meier method. Postoperative complications were defined and graded according to the Clavien–Dindo classification [10]. Postoperative pancreatic fistula [11] and delayed gastric emptying [12] were defined based on the International Study Group of Pancreatic Fistula (ISGPF) criteria.

### 2.2 Treatment Planning and Protocol of CIRT for PDAC

Carbon‐ion beams with energies of 290, 380, or 400 MeV/u were generated using a heavy‐ion accelerator at the GHMC and selected according to the tumor depth. Treatment planning was performed using the XiO‐N system (version 4.47; Elekta AB, Stockholm, Sweden; Mitsubishi Electric, Tokyo, Japan), which incorporates an ion‐beam dose calculation engine (K2dose). Clinical doses are expressed as relative biological effectiveness (RBE)‐weighted doses, calculated by multiplying the physical dose by the RBE of carbon‐ion beams, based on a mixed‐beam model derived from human salivary gland tumor cell survival [13]. Before CIRT, patients were immobilized using custom‐made fixation cushions and thermoplastic shells, and planning, four‐dimensional, and contrast‐enhanced CT images were acquired. Carbon‐ion radiotherapy was delivered once daily, 4 days per week (Tuesday–Friday), under respiratory gating, with a single fixed beam port treated per session. All patients fasted for at least 3 h prior to treatment. In standard cases, the prescribed dose was 55.2 Gy in 12 fractions. Details of treatment planning were previously reported [8]. The dose distribution of CIRT is shown in Figure 3A,B. We always performed a second respiratory‐gated CT scan a day before the start of CIRT to confirm the dose distribution to the GTV and at‐risk organs and, if necessary, to create a new treatment plan. At the time of irradiation, the positions of the target and risky organs were matched using fluoroscopy or in‐room CT. If there was a large displacement, a new treatment plan was created based on the new CT. All patients received concurrent oral S‐1 during CIRT according to our institutional protocol.

### 2.3 Clinical Course and Surgical Procedure

Because of the retrospective nature of this study, treatment pathways were not predefined institutional protocols but rather represented the clinical courses that patients ultimately followed. Treatment strategies were determined through multidisciplinary cancer board discussions according to individual clinical circumstances, including disease extent, treatment timing, response to preceding therapy, patient condition, and patient preference. Accordingly, for the purpose of this retrospective analysis, patients were categorized into two treatment courses according to the sequence of therapies they ultimately received. First, patients with advanced pancreatic cancer (BR‐A or UR‐LA) underwent CIRT with chemotherapy, and surgical resection was considered when the tumor was deemed technically resectable based on imaging findings and biologically resectable according to both tumor markers and FDG‐PET evaluation. Second, patients initially received systemic chemotherapy for advanced pancreatic cancer, after which CIRT was administered once surgical resectability was confirmed using the same imaging and biological markers, followed by surgical resection. Surgical indications were determined based on improvement in biological tumor markers and FDG‐PET and confirmation of CY0/M0 disease at staging laparoscopy.

After confirming the absence of peritoneal spread and distant metastases, curative‐intent pancreatic resection was performed according to the tumor location, consisting of pancreatoduodenectomy or distal pancreatectomy with regional lymph node dissection. When required, combined vascular resection was performed, with or without vascular reconstruction, depending on anatomical considerations. Venous reconstruction involving the portal vein, superior mesenteric vein, or confluence was primarily achieved through end‐to‐end anastomosis. For selected cases, arterial resection was incorporated en‐bloc, including celiac axis resection, as part of distal pancreatectomy (DP‐CAR). As a general principle, combined resection of the superior mesenteric artery (SMA) was not performed. For tumors showing abutment or suspected invasion around the SMA, dissection was performed with the anticipated therapeutic effect of CIRT, with the aim of preserving the perivascular nerve plexus as much as possible. However, in a limited number of cases, technical difficulties necessitated partial exposure of the arterial adventitia. All procedures were performed by experienced surgeons who had performed more than 100 cases of pancreatic cancer surgery.

### 2.4 Evaluation of Histological Findings and Therapeutic Response After Carbon‐Ion Chemoradiotherapy

Two board‐certified pathologists independently evaluated the resected specimens. The histological subtype, tumor differentiation, microscopic vascular invasion, and TNM staging were assessed according to standard pathological criteria. To evaluate the histological therapeutic effect of CIRT combined with chemotherapy, treatment response was graded using the Evans grading system [14], College of American Pathologists (CAP) tumor regression grade (Amin MB, et al. AJCC Cancer Staging Manual, 8th edition), and the Japanese Pancreatic Cancer Classification (General Rules for the Study of Pancreatic Cancer; Japanese Pancreas Society).

### 2.5 Statistical Methods

Continuous data are presented as the mean (standard deviation [SD]) or median (range). Differences between the two cohorts were assessed using Fisher's exact test and the Mann–Whitney *U* test, as appropriate. PFS and OS after initial treatment and RFS and OS after surgical resection were estimated using the Kaplan–Meier method. Statistical significance was determined using the log‐rank test to compare two groups. An exploratory correlation analysis was performed to evaluate the relationship between the interval from completion of CIRT to surgery and operative time using Spearman's rank correlation coefficient. Statistical significance was defined as *p* < 0.05. Statistical analyses were performed using JMP software version 15.0 (SAS Inc., Chicago, IL, USA).

## 3 Results

### 3.1 Preoperative Treatment Course Prior to Surgical Resection

The median duration from initiation of initial treatment to surgical resection was 7.5 months (5.5–8.7) in patients with BR‐A and 15.5 months (6.7–21.9) in patients with UR‐LA disease. The median interval from completion of carbon‐ion chemoradiotherapy to surgery was 5.6 months (0.9–14.8). Induction chemotherapy consisted of GnP in 7 patients (58%), modified FOLFIRINOX in 3 patients (25%), and a sequential regimen of GnP followed by modified FOLFIRINOX in 2 patients (17%). The median postoperative follow‐up duration after initial treatment and after surgery was 31.3 months (13.1–90.1) and 21.2 months (6.3–67.2), respectively.

The baseline characteristics of the study population are summarized in Table 1. The patients were divided into those treated with CIRT alone (*n* = 80) and those who underwent resection following CIRT (*n* = 12). There were no significant differences between the two groups in terms of age, sex distribution, body mass index, tumor location, or tumor marker levels. Patients undergoing resection following CIRT constituted a highly selected subgroup with more favorable nutritional and biological profiles, as evidenced by significantly higher serum albumin levels (4.4 vs. 3.9 g/L, *p* < 0.001) and initial prognostic nutritional index (52.5 vs. 45.1, *p* = 0.002) than those treated with CIRT alone. Tumor size was also significantly larger in the CIRT + resection group (median 31 mm vs. 24 mm, *p* = 0.023). In the resection group, the median PNI decreased from 52.5 (range: 45.2–54.9) at initial presentation to 45.3 (33.2–56.5) before surgery. Surgical resection after CIRT was technically feasible in all patients, although marked fibrosis was more frequently observed in patients with longer intervals between CIRT and surgery. An exploratory analysis demonstrated a moderate positive correlation between the interval from completion of CIRT to surgery and operative time (Spearman's *ρ* = 0.497, *p* = 0.101) (Figure S1). Surgical procedures included pancreaticoduodenectomy in five patients (42%), distal pancreatectomy in two (17%), and distal pancreatectomy with celiac axis resection (DP‐CAR) in five patients (42%). Arterial resection was required in five patients (42%) and portal vein resection in two patients (17%). No Grades 4 or 5 treatment‐related adverse events associated with CIRT plus S‐1 were observed, whereas Grade 3 neutropenia occurred in 2 of 12 patients. No patient became ineligible for surgery because of treatment‐related toxicity. In addition, no apparent association was observed between the induction chemotherapy regimen and postoperative complications.

**TABLE 1:** Baseline characteristics of patients treated with CIRT with or without subsequent resection.

Variables	CIRT (*n* = 80)	CIRT + resection (*n* = 12)	*p*
Age, years, mean ± SD	66 ± 9	68 ± 10	0.606
Sex, male:female	47:33	8:4	0.760
BMI, kg/m^2^, mean ± SD	22.2 ± 3.7	22.8 ± 2.6	0.291
*Location of tumor, number (%)*			
Pancreas head/uncus	36 (45)	5 (42)	0.828
Pancreas body	44 (55)	7 (58)	
*Laboratory data*			
Albumin, g/L	3.9 (2.5–4.5)	4.4 (3.9–4.7)	< 0.001
Creatinine	0.7 (0.4–17.0)	0.8 (0.5–1.0)	0.067
Initial PNI, median (range)	45.1 (28.6–61.9)	52.5 (45.2–54.9)	< 0.001
Preoperative PNI, median (range)	—	45.3 (33.2–56.5)	—
*Tumor markers, median (range)*			
CEA	3.3 (0.6–16.9)	3.0 (0.8–10.5)	0.706
CA19‐9	183 (0–7852)	146 (4–2443)	0.926
DUPAN‐2	120 (25–4183)	50 (25–6493)	0.749
Tumor size, mm (range)	24 (8–56)	31 (24–41)	0.023
*Resectability, number (%)*			
BR‐A	11 (14)	3 (25)	0.384
UR‐LA	69 (86)	9 (75)	

Abbreviations: BMI, body mass index; BR‐A, borderline resectable with arterial involvement; CA19‐9, carbohydrate antigen 19‐9; CEA, carcinoembryonic antigen; CIRT, carbon‐ion radiotherapy; DUPAN‐2, duodenal pancreatic cancer antigen; PNI, prognostic nutritional factor; UR‐LA, unresectable, locally advanced.

### 3.2 Surgical Outcomes and Histological Findings

The surgical and pathological outcomes of the 12 patients who underwent pancreatic resection after CIRT are summarized in Table 2. The median operative time was 489 min (range: 258–660 min), and the median intraoperative blood loss was 364 mL (range: 79–1205 mL). Intraoperative blood transfusion was required in one patient (8%). Postoperative complications included pancreatic fistula of ISGPF Grade ≥ B in one patient (8%), intra‐abdominal abscess in two (17%), and chylous leakage in three (25%). Major postoperative complications of Clavien–Dindo Grade ≥ IIIa occurred in three patients (25%). No postoperative mortality occurred, and the median postoperative hospital stay was 16 days (range: 12–57 days).

**TABLE 2:** Surgical outcomes and histological findings in patients who underwent resection following CIRT.

Variables	CIRT + resection (*n* = 12)
*Surgical procedure, number (%)*	
PD	5 (42)
DP	7 (58)
*Combined resection of vessels, number (%)*	
Celiac artery	5 (42)
Portal vein	3 (25)
Operative time, min. (range)	489 (258–660)
Blood loss, mL (range)	364 (79–1205)
Intraoperative blood transfusion, number (%)	1 (8)
*Postoperative complication, number (%)*	
Pancreatic fistula (ISGPF Grade ≥ B)	1 (8)
Intraabdominal abscess	2 (17)
Chylous leakage	3 (25)
Clavien‐Dindo Grade ≥ IIIa	3 (25)
Length of hospital stay after surgery, days, median (range)	16 (12–57)
Mortality, number (%)	0 (0)
*Pathological findings*	
Lymph node metastasis, number (%)	0 (0)
Number of harvested lymph node, median (range)	13 (4–28)
R0 resection, number (%)	11 (92)
*Therapeutic effect (EVANS grade), number (%)*	
Grade I	1 (8)
Grade IIa	1 (8)
Grade III	7 (58)
Grade IV	3 (25)
*Therapeutic effect (CAP grade), number (%)*	
Grade 3	2 (17)
Grade 2	0 (0)
Grade 1	7 (58)
Grade 0	3 (25)
*Therapeutic effect (JPS), number (%)*	
Grade 1b	2 (17)
Grade 2b	1 (8)
Grade 3	6 (50)
Grade 4	3 (25)

Abbreviations: CAP, College of American Pathologists; CIRT, carbon‐ion radiotherapy; DP, distal pancreatectomy; ISGPF, International Study Group of Pancreatic Fistula; JPS, Japanese Pancreatic Society; PD, pancreaticoduodenectomy.

Pathological examination revealed no lymph node metastases in any patient, with a median of 13 harvested lymph nodes (range: 4–28). R0 resection was achieved in 11 of the 12 patients (92%). According to the Evans classification, the histological therapeutic response was graded as Grade I in one patient (8%), Grade IIa in one (8%), Grade III in seven (58%), and Grade IV in three (25%). Overall, a marked histological response (Evans Grades III–IV) was observed in ten of the 12 patients (83%). Using the College of American Pathologists (CAP) grading system, Grades 3, 1, and 0 were observed in two (17%), seven (58%), and three (25%) patients, respectively. According to the Japanese Pancreatic Society classification, Grades 1b, 2b, 3, and 4 were identified in two (17%), one (8%), six (50%), and three (25%) patients, respectively.

### 3.3 Changes in Tumor Size During Treatment

Changes in tumor size during the course of treatment are shown in Figure 1. Tumor size was compared among the initial clinical tumor size (initial cTS), preoperative clinical tumor size (preoperative cTS), and pathological tumor size (pTS) in all 12 patients. A significant reduction in tumor size was observed between the initial and preoperative cTS (*p* = 0.004). Furthermore, the pathological tumor size was significantly smaller than that of the preoperative cTS (*p* = 0.019). These findings indicate a stepwise decrease in tumor size from initial clinical assessment to pathological evaluation.

**FIGURE 1:**
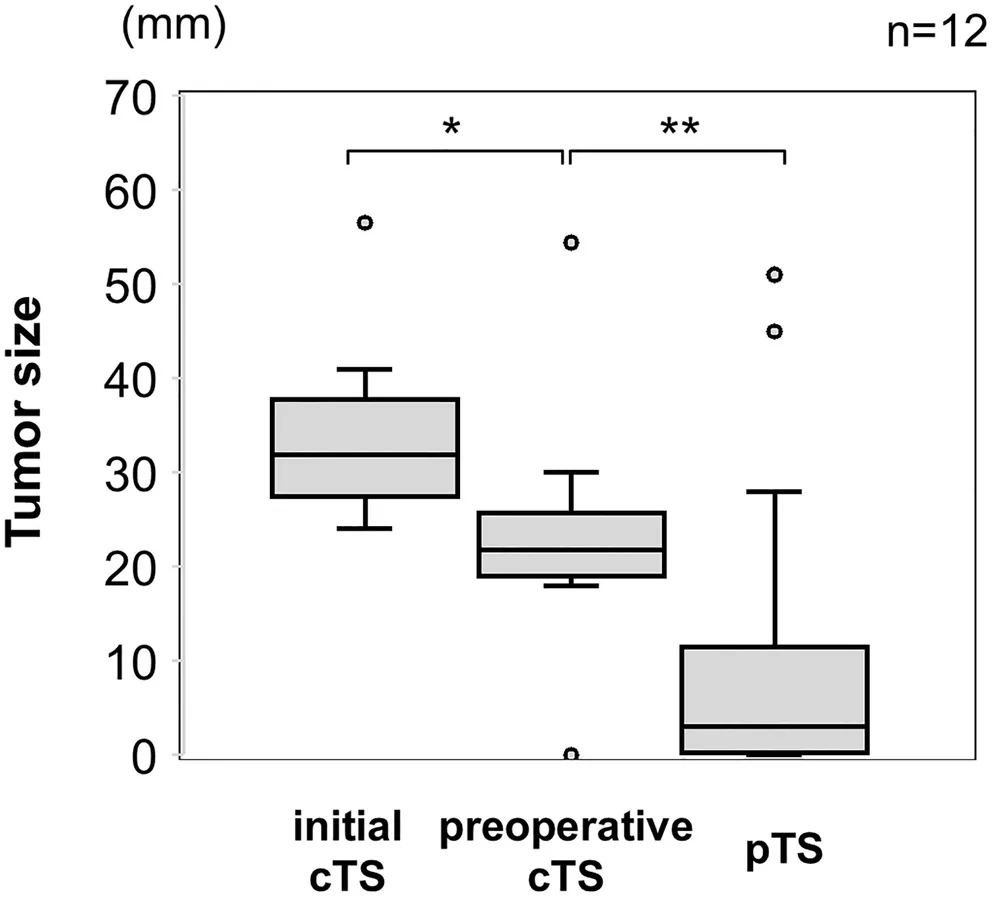
Comparison of tumor size between initial clinical tumor size (cTS), preoperative cTS, and pathological tumor size (pTS). Tumor size (mm) was compared among the initial clinical assessment (initial cTS), preoperative clinical assessment (preoperative cTS), and pathological tumor size (pTS). Data are presented for the patients with available measurements (*n* = 12). A single asterisk (*) indicates a statistically significant difference (*p* = 0.004) and a double asterisk (**) indicates a significant difference (*p* = 0.019).

### 3.4 Oncological Outcomes After Initial Treatment and After Surgical Resection

The 3‐year PFS and OS rates calculated from the initiation of treatment (start of induction chemotherapy) were 74.1% and 79.5%, respectively (Figure 2A,B). When survival was evaluated from the date of surgical resection, the 3‐year RFS and OS rates were 47.6% and 50.8%, respectively (Figure 2C,D). Postoperative recurrence occurred in five patients (42%). The sites of recurrence included liver metastasis in 1 patient (8%), lung metastasis in one patient (8%), lymph node metastasis outside the local region in two patients (17%), and peritoneal dissemination in two patients (17%). None of the patients developed local recurrence within the CIRT irradiation field. At the time of analysis, the median survival time (MST) for overall survival was not reached when calculated from either the initiation of treatment or the date of surgical resection.

**FIGURE 2:**
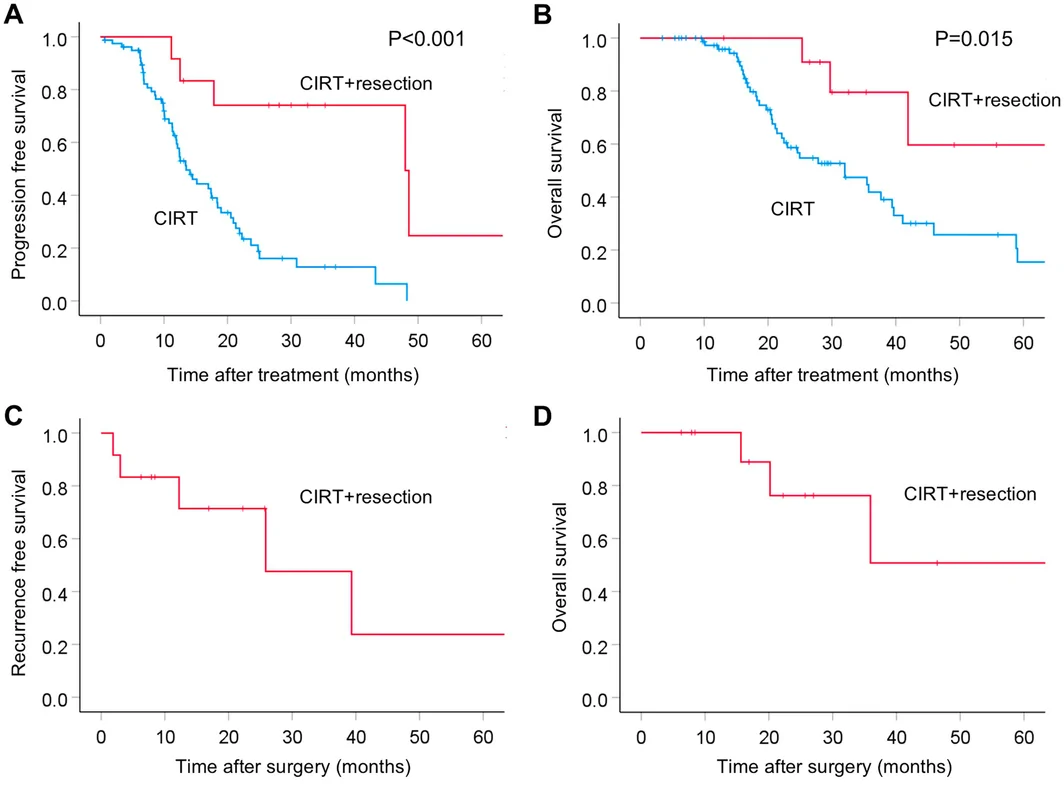
Kaplan–Meier survival curves according to treatment strategy following carbon‐ion radiotherapy (CIRT). (A) Progression‐free survival (PFS) calculated from treatment initiation (start of induction chemotherapy). (B) Overall survival (OS) calculated from treatment initiation. (C) Recurrence‐free survival (RFS) calculated from the date of surgical resection. (D) Overall survival (OS) calculated from the date of surgical resection. Panels (A, B) compare patients who underwent surgical resection following CIRT (CIRT + resection, *n* = 12) with those treated with CIRT combined with chemotherapy without subsequent resection (CIRT alone, *n* = 80).

For comparison, patients with BR‐A/UR‐LA pancreatic cancer treated with CIRT without subsequent surgical resection were also analyzed. The 3‐year PFS and OS rates calculated from treatment initiation were 12.5% and 41.9%, respectively, in the CIRT‐alone group, compared to 74.1% and 79.5%, respectively, in patients who underwent resection following CIRT (Figure 2A,B). Exploratory analysis demonstrated a significantly longer PFS in the resection group than in the CIRT‐alone group (*p* < 0.001). OS was also significantly improved in patients who underwent resection (*p* = 0.015). The median follow‐up duration of the entire cohort was 32.7 months.

### 3.5 Representative Case

Figure 3 shows a representative case of a successful conversion surgery following CIRT‐based multimodal treatment. A 76‐year‐old man was diagnosed with UR‐LA of the pancreatic body (Figure 3C). Induction chemotherapy with gemcitabine plus nab‐paclitaxel was administered for 5 months, followed by CIRT at a total dose of 55.2 Gy, and modified FOLFIRINOX for 9 months. During treatment, marked tumor shrinkage (Figure 3D) and biological response were observed, with a substantial decrease in tumor markers (CEA: 9.0–1.7 ng/mL; CA19‐9: 984–5 U/mL; DUPAN‐2: 465 to ≤ 25 U/mL) and a reduction in metabolic activity on FDG‐PET (max SUV from 15.0 to 2.4). Twenty‐two months after treatment initiation, DP‐CAR was performed for conversion surgery (Figure 3E). Histopathological examination demonstrated extensive fibrosis with only sparse residual atypical glands, corresponding to Evans Grade III therapeutic response (Figure 3F). The patient remains alive without recurrence at 90.1 months after the initial treatment and 67.2 months after surgery.

**FIGURE 3:**
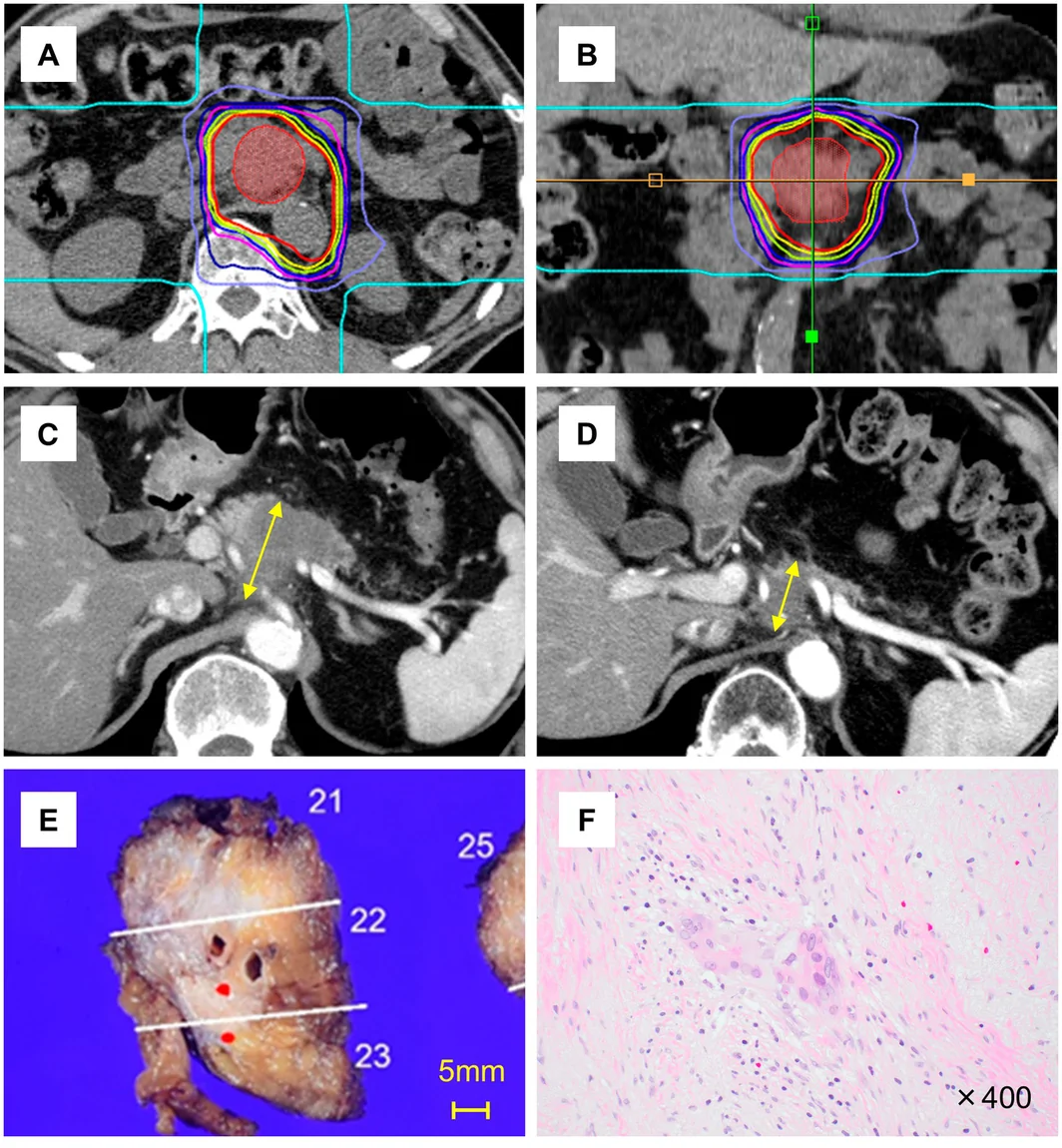
Representative case of conversion surgery following carbon‐ion radiotherapy (CIRT)‐based multimodality treatment. (A) Axial dose distribution of CIRT. The prescribed dose was 55.2 Gy (relative biological effectiveness [RBE]). Highlighted are 95% (red), 90% (yellow), 80% (light green), 70% (blue), 60% (pink), 50% (deep blue), 30% (light purple), and 10% (cyan) isodose lines. The region shown in red indicates the gross tumor volume. (B) Coronal dose distribution of CIRT. The yellow double‐headed arrows indicate the tumor diameter. (C) A CT image at initial diagnosis. (D) Preoperative CT images after induction chemotherapy and CIRT. (E) Resected specimen after distal pancreatectomy with celiac axis resection. (F) Histopathological findings of the resected tumor (H&E staining, ×400).

## 4 Discussion

In the present study, pancreatic resection following CIRT‐based multimodal treatment was performed safely with acceptable perioperative morbidity, even in patients with arterial involvement. Although treatment‐related fibrosis was frequently encountered, surgical dissection remained technically feasible, resulting in a high R0 resection rate and no local recurrence within the irradiation field. Although excessive tissue fibrosis related to CIRT may raise concerns regarding technical difficulties, our data indicate that pancreatectomy following CIRT was safely performed with acceptable morbidity, comparable to standard pancreatectomy. These findings are consistent with the phase I trial for resectable stage reported by Shinoto et al. [9], which demonstrated that preoperative CIRT followed by surgery for resectable pancreatic cancer was feasible and tolerable without unacceptable morbidity, achieving excellent local control. In addition, a few case reports have recently described conversion surgery following chemotherapy and radical‐dose CIRT in initially unresectable locally advanced PDAC, providing preliminary clinical insight into the feasibility of surgery after CIRT, although robust evidence is still lacking [15]. Notably, in our experience, a longer interval between CIRT and surgery tended to be associated with more pronounced fibrosis and tissue hardening, which may increase the technical difficulty of dissection. Exploratory analysis demonstrated a moderate positive correlation between the interval from CIRT completion to surgery and operative time (Spearman's *ρ* = 0.497, *p* = 0.101). Although this finding may support our intraoperative observations, it should be interpreted cautiously because of the small sample size and heterogeneity of surgical procedures. Therefore, from a surgical perspective, prolonged delay after completion of CIRT should preferably be avoided when surgical resection is anticipated. However, the optimal interval between completion of CIRT and surgery remains to be determined, and treatment timing should be individualized while balancing oncological assessment and operative safety.

Accumulating evidence suggests that CIRT provides meaningful local tumor control with acceptable toxicity in patients with pancreatic cancer, even in clinically challenging populations. Previous studies have demonstrated favorable outcomes of CIRT in elderly patients who are often considered poor candidates for aggressive surgery, indicating its robustness as a local treatment modality [16]. More recently, a multicenter prospective phase II study evaluating preoperative chemo‐CIRT for resectable and borderline resectable pancreatic cancer reported encouraging safety and oncological outcomes, further supporting the feasibility of integrating CIRT into multimodal strategies [17]. Given the strong local control achieved by CIRT and its ability to suppress microscopic perivascular extension, CIRT‐based multimodality treatment is conceptually more suited to PDAC with arterial involvement (BR‐A or UR‐LA), where local disease control and margin‐negative resection remain the principal barriers to conversion surgery. In this context, the role of CIRT should be viewed not merely as an alternative to surgery but as a key enabling component of multidisciplinary treatment aimed at achieving R0 resection, potentially offering greater benefit in BR‐A/UR‐LA disease than in primarily resectable pancreatic cancer.

Compared with conventional X‐ray‐based chemoradiotherapy (CRT), CIRT has demonstrated superior local control and survival outcomes in patients with locally advanced pancreatic cancer. In conventional CRT, the reported median OS is approximately 14–17 months, with a 2‐year local control rate of around 39%–42% [18]. In contrast, a recent CIRT series reported a median survival of 29.6 months and a 2‐year local control rate of 76.1%, even in unresectable disease [8]. These results suggest that CIRT provides more effective local disease control than conventional CRT, which may translate into improved survival when integrated into multimodality treatment strategies. A recent study evaluating surgical resection after high‐dose proton beam therapy (PBT) reported an R0 resection rate of 91%, pathological complete response in 27% of patients, and a median overall survival of 52.9 months [19]. Notably, no local recurrence within the CIRT irradiation field occurred, whereas local recurrence was reported in three patients after PBT‐based treatment. CIRT possesses a higher RBE and linear energy transfer than PBT, resulting in distinct radiobiological characteristics. However, direct clinical comparisons between CIRT and PBT in pancreatic cancer are currently lacking, and further studies are required to clarify the relative clinical advantages of these particle beam modalities.

Importantly, the histopathological tumor response was remarkable, with EVANS Grades III–IV response observed in 83% of the patients, which may partly explain the high R0 resection rate and favorable survival outcomes observed in this cohort. Therefore, CIRT may represent a strong local control modality that facilitates conversion surgery in selected patients with initially unresectable disease. Previous studies have reported moderate rates of marked histological responses in patients with pancreatic cancer treated with BR‐A or UR‐LA using intensive neoadjuvant approaches and conversion surgery after chemoradiotherapy. Evans Grades III–IV responses were observed in 32% of patients in a TNT cohort [20] and 32% (Grade III: 17%, Grade IV: 15%) in a CRT‐based conversion surgery series [3]. Other CRT‐based approaches for BR‐A/UR‐LA pancreatic cancer have reported Evans Grades III–IV response rates ranging from less than 10% to approximately 40%, depending on patient selection and treatment intensity [21, 22]. In contrast, our cohort demonstrated a high frequency of Evans Grades III–IV response (83%) in BR‐A/UR‐LA pancreatic cancer, with no pathological lymph node metastasis observed, suggesting that CIRT may provide more potent local tumor control than conventional CRT within intensive neoadjuvant or conversion‐oriented treatment paradigms. This finding is noteworthy because BR‐A and UR‐LA pancreatic cancer is generally associated with a high incidence of pathological lymph node metastasis. As the CIRT field included the regional lymph node area, this observation may partly reflect the strong locoregional treatment effect of CIRT‐based multimodal therapy. However, patient selection and the limited sample size should also be considered when interpreting these findings.

In the present exploratory analysis, patients who underwent surgical resection following CIRT‐based multimodality treatment demonstrated significantly prolonged PFS and OS compared to those treated with CIRT combined with chemotherapy alone. These findings suggest that, in selected patients, successful conversion to surgical resection after CIRT may contribute not only to improved disease control but also to enhanced long‐term survival outcomes. The observed survival benefit may be explained by the strong local tumor control achieved with CIRT, whereas subsequent surgical resection enables definitive removal of residual viable disease. However, the conversion surgery rate was 13.0% (12/92), and only patients demonstrating favorable biological and radiological responses proceeded to surgery. Although indications for CIRT and subsequent surgical resection were determined through multidisciplinary evaluation, treatment selection and subsequent surgical decision‐making were influenced by multiple oncological and patient‐related factors. The non‐resected cohort was therefore heterogeneous and should not be regarded as a uniform population of patients who were eligible for surgery but simply did not undergo resection. Consequently, the Kaplan–Meier comparison between resected and non‐resected patients should be regarded as exploratory rather than a definitive comparison of treatment efficacy. Importantly, despite the absence of surgical resection, survival outcomes in the non‐resected CIRT cohort were comparable to, or appeared slightly more favorable than, those reported for non‐surgical treatment modalities, including chemotherapy alone or conventional chemoradiotherapy, in patients with BR‐A and UR‐LA pancreatic cancer [3, 20, 23].

Therefore, these findings should be interpreted with caution. Although our results suggest a potential oncological benefit of surgical resection following CIRT, this analysis remains exploratory and hypothesis‐generating. Further prospective studies are needed to confirm the survival impact of this strategy. Several studies have investigated the relationship between the histopathological therapeutic response and prognosis in pancreatic cancer. Zhao et al. [24] identified pathological complete response (pCR) as a favorable prognostic factor. Chatterjee et al. [25] reported that defining a favorable response as an estimated residual tumor rate of ≤ 10% was associated with improved prognosis, whereas no prognostic difference was observed when a cutoff of 50% was applied. In contrast, Murata et al. [26] demonstrated a significant prognostic difference using a 50% residual tumor cutoff. Furthermore, White et al. [27] reported that an estimated residual tumor rate of ≥ 90% was associated with poor prognosis. Although the available evidence regarding correlations between the degree of histopathological response and various clinical outcomes remains limited and somewhat inconsistent, achieving a favorable histopathological response—including pCR—can reasonably be expected to exert a positive impact, particularly with respect to R0 resection and durable local control, within the context of multimodal treatment strategies for pancreatic cancer. There have also been reports highlighting a discrepancy between radiographic assessment and histopathological response after neoadjuvant treatment for pancreatic cancer, suggesting that reassessment after NAT should consider the treatment modality (chemotherapy vs. chemoradiotherapy) and should not rely solely on imaging findings but rather integrate pathological and biological factors when determining surgical indication [28].

Pathological specimens obtained after pancreatic resection following neoadjuvant therapy represent a valuable source of information, enabling accurate assessment of the extent of tumor cell destruction induced by radiotherapy including CIRT or chemotherapy, and the degree of residual cancer cells resistant to treatment. Serum tumor markers are insufficient to fully capture the disease status or therapeutic response. Accordingly, histopathological assessment of the therapeutic response after neoadjuvant treatment plays a critical role and has been described by the Union for International Cancer Control as a new and promising prognostic factor. In this study, the histopathological therapeutic response was evaluated using the Evans classification, CAP grading system, and criteria defined in the Japanese Pancreatic Cancer Classification. One of the major limitations of the current situation is that multiple grading systems coexist, each with different evaluation items and criteria, making the interpretation and comparison of results difficult. In addition, the reproducibility and interobserver agreement of histopathological response assessments have been reported to be suboptimal [29]. In pancreatic cancer, fibrotic changes frequently develop in areas where tumor cells are present before treatment, making it challenging to distinguish residual tumors from therapy‐induced fibrosis and accurately delineate the true tumor extent. Establishment of a standardized, reliable, and universally applicable histopathological response assessment system for pancreatic cancer is highly anticipated.

The limitations of this study include its retrospective design, small sample size, and potential for patient selection bias. In addition, this analysis was conducted at a single center, which may limit the generalizability of the findings. This study included a highly selected cohort of patients who successfully underwent conversion surgery after CIRT‐based multimodal treatment. Therefore, the favorable outcomes observed may have been influenced not only by treatment effects but also by patient selection and institutional expertise. Furthermore, the heterogeneity in preoperative treatment strategies, including differences in treatment sequence and the interval between CIRT and surgery, may have influenced pathological response, surgical complexity, and oncological outcomes. Nevertheless, to the best of our knowledge, this is the first case series of surgical resection following CIRT in patients with BR‐A or UR‐LA pancreatic cancer, suggesting that a CIRT‐based treatment strategy may be a promising component of future multidisciplinary approaches. Based on these findings, we initiated a prospective phase I/II clinical trial (jRCTs 031230335) on surgical resection following CIRT for BR‐A/UR‐LA pancreatic cancer, and further results from this ongoing study are anticipated.

In conclusion, surgical resection following CIRT‐based multimodal treatment was feasible and safe, achieving favorable histopathological responses and durable local control in selected patients with PDAC and arterial involvement. These findings support the potential role of CIRT in facilitating conversion surgery for advanced pancreatic cancer; however, further validation in larger prospective studies is warranted.

## Author Contributions

**Hayato Ikota:** methodology, formal analysis, validation, investigation, writing – original draft, writing – review and editing. **Norio Kubo:** methodology, formal analysis, data curation, writing – original draft, investigation, validation, writing – review and editing. **Mariko Tsukagoshi:** resources, data curation, validation. **Masahiko Okamoto:** data curation, validation, investigation, formal analysis, writing – original draft, writing – review and editing. **Yuhei Miyasaka:** data curation, resources, writing – original draft, methodology, funding acquisition, visualization, writing – review and editing. **Ken Shirabe:** supervision, writing – original draft, conceptualization, funding acquisition, writing – review and editing. **Ryo Muranushi:** data curation, resources, validation. **Takamichi Igarashi:** data curation, formal analysis. **Kenichiro Araki:** conceptualization, methodology, data curation, writing – review and editing, writing – original draft, investigation, validation, formal analysis, funding acquisition, visualization, project administration, resources. **Tatsuya Ohno:** writing – original draft, conceptualization, methodology, supervision, funding acquisition, writing – review and editing.

## Funding

This work was supported in part by the 2022 Cancer Clinical Research Grant Program of the Japan Society of Clinical Oncology.

## Conflicts of Interest

Ken Shirabe, who is a co‐author of this article, is an editor in chief in the Annals of Gastroenterological Surgery. The authors declare no other conflicts of interest for this article.

## Supporting information


**Figure S1:** Correlation between the interval from completion of carbon‐ion radiotherapy (CIRT) and operative time. An exploratory Spearman rank correlation analysis demonstrated a moderate positive correlation (*ρ* = 0.497, *p* = 0.101).

## Data Availability

The data that support the findings of this study are available on request from the corresponding author. The data are not publicly available due to privacy or ethical restrictions.
