# Supplementary material for: Impact of Histopathological Response on Outcomes After Surgical Resection Following Carbon‐Ion Radiotherapy for Pancreatic Cancer With Arterial Involvement
Source: Ann Gastroenterol Surg. 2026 Aug 2:10.1002/ags3.70259. Online ahead of print. doi: 10.1002/ags3.70259 (PMC13429861; doi:10.1002/ags3.70259)
Supplement: Supplementary file 1 — Figure S1: Correlation between the interval from completion of carbon‐ion radiotherapy (CIRT) and operative time. An exploratory Spearman rank correlation analysis demonstrated a moderate positive correlation (ρ = 0.497, p = 0.101). [file AGS3-9999-0-s001.docx]

Supporting Information

**Supplementary Figure Legends**

**Supplementary Figure 1.**

Correlation between the interval from completion of carbon-ion radiotherapy (CIRT) and operative time. An exploratory Spearman rank correlation analysis demonstrated a moderate positive correlation (ρ = 0.497, P = 0.101).
